# Quantifying the Evidence for the Risk of Metabolic Syndrome and Its Components following Androgen Deprivation Therapy for Prostate Cancer: A Meta-Analysis

**DOI:** 10.1371/journal.pone.0117344

**Published:** 2015-03-20

**Authors:** Cecilia Bosco, Danielle Crawley, Jan Adolfsson, Sarah Rudman, Mieke Van Hemelrijck

**Affiliations:** 1 King’s College London, School of Medicine, Division of Cancer Studies, Cancer Epidemiology Group, London, United Kingdom; 2 Department of Oncology, Guy’s & St Thomas’ NHS Foundation Trust, London, United Kingdom; 3 Karolinska Institute, CLINTEC Department, Stockholm, Sweden; Innsbruck Medical University, AUSTRIA

## Abstract

**Background:**

No meta-analysis is yet available for the risk of metabolic syndrome (MetS) following androgen deprivation therapy (ADT) for men with prostate cancer. To summarize the evidence for the link between ADT and MetS or its components quantitatively with a meta-analysis including all studies published to date.

**Methods:**

PubMed and Embase were searched using predefined inclusion criteria to perform meta-analyses on the association between metabolic syndrome, hyperglycemia, diabetes, hypertension, dyslipidemia or obesity and androgen deprivation therapy in patients with prostate cancer. Random effects methods were used to estimate pooled relative risks (RRs) and 95% confidence intervals (CI).

**Results:**

A total of nine studies was included. There was a positive association between ADT and risk of MetS (RR: 1.75 (95% CI: 1.27–2.41)). Diabetes was the only MetS component present in more than 3 studies, and also showed an increased risk following ADT (RR: 1.36 (95% CI: 1.17–1.58)).

**Conclusion:**

This is the first quantitative summary addressing the potential risk of MetS following ADT in men with PCa. The positive RRs indicate that there is a need to further elucidate how type and duration of ADT affect these increased risks of MetS and diabetes as the number of men with PCa treated with ADT is increasing.

## Introduction

Androgen deprivation therapy (ADT), which interrupts testosterone regulation of the prostate tumour, has been the cornerstone treatment for men with locally advanced or metastatic prostate cancer (PCa) since the 1940’s [1]. It is a highly effective treatment that inhibits testosterone production rendering patients medically castrated. A number of side effects have been reported including osteoporosis, sexual dysfunction, anaemia, cardiovascular and thromboembolic disease, and metabolic changes such as weight gain, diabetes, insulin resistance, and dyslipidemia [2–10]. The latter symptoms are all part of the metabolic syndrome (MetS), which is suggested to be a common side effect for PCa men treated with ADT [11–13]. Metabolic syndrome is defined by a combination of metabolic risks: increased waist circumference (visceral obesity), raised triglycerides or its specific treatment, reduced high-density lipoprotein cholesterol or its specific treatment, raised blood pressure or treatment of previously diagnosed hypertension, and raised fasting plasma glucose or its specific treatment. The joint statement of major international associations [14] defines everybody with three of the above listed metabolic risks as having MetS.

However, to our knowledge there is no meta-analysis to date quantifying the potential association between ADT and MetS in men with PCa [11,13,15,16]. Moreover, the underlying mechanisms are not well understood. Some studies demonstrated that a decrease in testosterone levels is associated with a decrease of 2.7–3.8% in lean body mass and an increase of 9.4–11.0% in fat mass [17–19]. The same studies indicated that ADT increases fasting plasma insulin levels [8,17,19]. Nonetheless, it is important to note that most studies of these metabolic risk factors were conducted in very small study populations over a very short follow-up period of six to twelve months [18,19]. So even though the results were statistically significant, larger prospective studies are required to confirm these findings.

In addition, there are some studies that suggest a different pathway between ADT and metabolic risk factors. For example, a small study showed that ADT preferentially increase subcutaneous rather than visceral abdominal fat and increase rather than decrease HDL cholesterol, which is in contrast with the traditionally described MetS [18]. Additionally, it was shown that ADT does not alter levels of C-reactive protein or other markers of inflammation, which suggests that ADT causes a pattern of metabolic changes that is distinct from the classically defined MetS [17].

Thus, it remains unclear to what extend ADT is associated with an increased risk of MetS or its components in men with PCa. In contrast to previous systematic reviews, this study aims to summarize the evidence for the link between ADT and MetS or its components quantitatively with a meta-analysis including all studies published to date.

## Methods

### Literature Search Strategy

We used computerized literature search databases (Pubmed search followed by an Embase and Cochrane Library search) to identify full-text and abstracts published to date. Our searches included “metabolic syndrome”, “hypertension”, “dyslipidemias”, “hyperglycemia”, “diabetes mellitus”, and “obesity” as search/Mesh terms for the exposure variable of interest. In addition, “prostatic neoplasms” and “androgen deprivation therapy” or “Antineoplastic Agents, Hormonal/adverse effects” were used as search/Mesh terms for the outcome variable of interest. Our search strategy was limited to publications with a focus on humans. By not restricting the search to research papers, we made it possible to include grey literature, such as letters and abstracts presented in relevant conference meetings to address the effects of ADT on MetS. In addition, all references of selected articles were checked, including hand searches, which are effective practical ways to cross-check the completeness of the electronic searches.

### Inclusion Criteria

The selected articles were chosen based on the following set of inclusion criteria: the publication pertained to an observational epidemiologic study which measured exposure to ADT; the comparison group was clearly defined as a different PCa population not on ADT; MetS or one of its components was assessed as an outcome; men with PCa were the main study population. The definition of a comparison group was important as several studies to date investigated the link between ADT and MetS by observing MetS before and after ADT in the same patients [8,17,19]. These findings may not necessarily reflect the effects of ADT as changes in the tumour itself can also have an effect on metabolic changes. These studies, often with a smaller sample size, were not included in the current meta-analysis as they did not contain control patients free of ADT. Mixing these studies with other observational studies using different control groups, would have resulted in a meta-analysis of less comparable studies.

**Fig 1:**
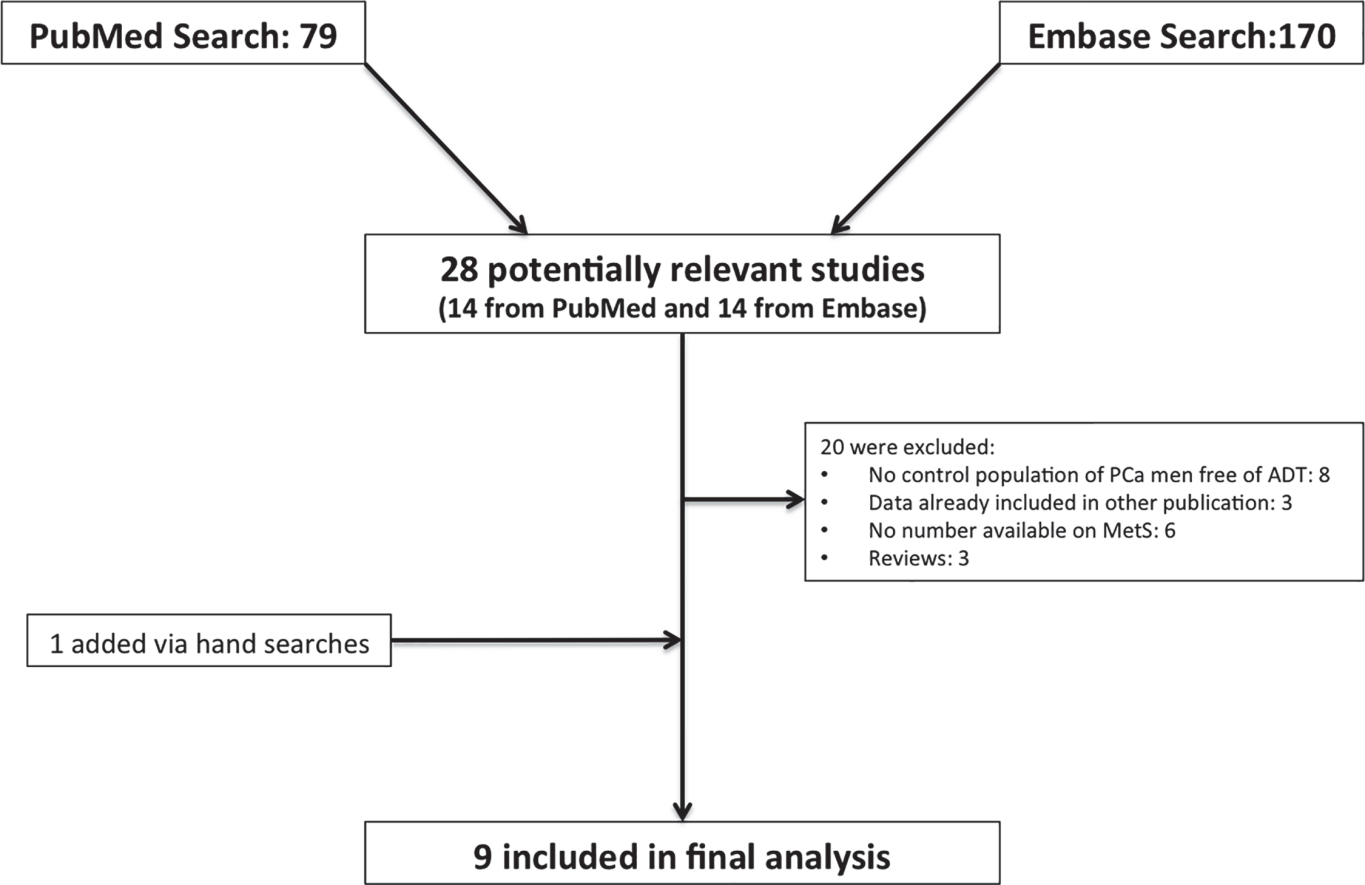
Flow chart of study selection for meta-analysis on ADT and MetS.

Body composition changes were not part of the current meta-analysis as a recent meta-analysis already showed that ADT has an immediate influence on body weight, BMI, percentage fat mass and percentage lean mass [20]. Initially, titles of articles were reviewed in order to ascertain whether they might potentially fit the inclusion criteria. If, after assessing the abstract, there was any doubt regarding whether it met the relevant criteria, it was kept for more thorough, subsequent assessment. The list of potential articles was further shortened by performing detailed evaluations of the methods and results of each remaining paper. Fig. 1 provides more detailed information regarding the progressive ‘flow’ of the study exclusion process. PRISMA checklist for systematic reviews and meta-analyses is provided in the S1 Checklist.

S2 Checklist shows how the STROBE criteria were used to evaluate the quality of included observational studies [21].

### Data Extraction

The following details were recorded for each study: author, year of publication, ADT exposure (binary), study type (case-control or cohort), outcome, and number of cases and total subjects for each level of ADT. The outcome was defined as MetS or any of its components: hyperglycemia, diabetes, hypertension, dyslipidemia, or obesity. Despite different definitions available, it is generally accepted that MetS is defined by a combination of metabolic risks: increased waist circumference (visceral obesity), raised triglycerides or its specific treatment, reduced high-density lipoprotein cholesterol or its specific treatment, raised blood pressure or treatment of previously diagnosed hypertension, and raised fasting plasma glucose or its specific treatment. The joint statement of major international associations defines everybody with three of the above listed metabolic risks as having MetS [14]. Hence, the current meta-analysis dichotomized the different outcomes irrespective of the definitions used.

### Meta-Analysis Statistical Techniques

The effect of ADT on risk of MetS or its components among men with PCa was evaluated by calculating the random effects summary relative risk. Forest plots were created as they display the relative risk estimates of MetS risk comparing both levels of ADT for each study. Potential heterogeneity of the study results was statistically evaluated using the I^2^ statistic. Potential publication bias was assessed using Begg’s Test. All analyses were performed using STATA (version 12).

## Results

The initial search for ADT and MetS or its components resulted in 79 articles via PubMed and 170 via Embase. After extracting information from the abstracts, 28 articles were selected for further investigation. Finally, nine studies were selected for primary data-analysis from which six studies were conducted in the United States, two in Spain, and one study in China. One additional study, conducted in Canada, was identified via hand searches (Fig. 1 and Table 1). Based on the above defined inclusion criteria we excluded 20 studies (Fig. 1). Amongst these, eight were excluded due to lack of control populations, three were systematic reviews, three presented data that overlapped with studies that were already included, and another six were omitted due to lack of information or methodological errors.

**Table 1:** Overview of studies included in meta-analyses.

Author	Country	Study Type	ADT Type	Outcome	Number of patients	Main findings
Keating et al. [37]	USA	Cohort	GnRH agonist, combined androgen blockage, orchiectomy, anti-androgens	Diabetes	14,597 ADT. 22,846 no ADT	No ADT: Ref. GnRH agonists: 1.28 (95%CI: 1.19–1.38) Orchiectomy: 1.16 (95%CI: 0.87–1.54) Combined androgen blockage: 1.17 (95%CI: 0.96–1.42). Oral anti-androgens: 1.02 (95%CI: 0.72–1.45)
Lage et al. [38]	USA	Retrospective claims database	Any ADT	Diabetes	1,231 on ADT. 7,250 no ADT	While controlling for other factors, the estimated relative risk of incident diabetes associated with the receipt of ADT was 1.36 (95%CI: 1.07–1.74)
Keating et al. [6]	USA	Cohort	GnRH agonist, orchiectomy	Diabetes	26,570 on ADT. 46,626 on ADT	No ADT: Ref. GnRH agonists: 1.44 (95%CI: 1.34–1.55) Orchiectomy: 1.34 (95%CI: 1.20–1.50)
Braga-Basaria et al.[39]	USA	Cross-sectional	ADT	Mets^1^ Obesity Hyperglycaemia Hypertriglyceridemia Low HDL Hypertension	20 on ADT 18 no ADT	Prevalence of MetS: 55% vs 22% (ADT vs no ADT). Prevalence of obesity: 75% vs 33% Prevalence of hyperglycemia: 65% vs 16%. Prevalence of hypertriglyceridemia: 55% vs 44%. Prevalence of low HDL: 35% vs 50%. Prevalence of hypertension: 45% vs 28%
Basaria et al.[40]	USA	Cross-sectional	ADT	Hyperglycaemia	18 on ADT 17 no ADT	Men on ADT had significantly higher levels of fasting serum glucose (131.0 mg/dL) compared with men not on ADT (103.0 mg/dL; P: 0.01)
Bo et al. [41]	China	Cross-sectional	ADT Orchiechtomy	Metabolic changes^2^	46 orchiechtomoy/ ADT 37 prostatechtomy no ADT. 50 controls.	After 3 months ADT group had increased levels of fasting serum insulin and LDL compared to the other 2 groups (P< 0.05) After 12 months ADT group had increased levels of waist circumference, fasting serum insulin and glucose, total cholesterol, HDL and LDL compared to the other 2 groups (P<0.05)
Garcia et al.[42]	Spain	Cross-sectional	ADT	Mets^1^. Osteoporosis	216 on ADT. 50 no ADT	Prevalence of Mets in no ADT: 19% Prevalence Mets ADT: 6 months treatment: 21%. 12–18 months treatment: 36%. >24 months treatment: 24%
Valverde et al.[43]	Spain	Cross-sectional	ADT	Mets^1^	53 on ADT. 104 no ADT (52 PCa 52 no PCa)	Mets in patients on ADT: 51.9%. Mets in patients without ADT: 35.8%
Alibhai et al. [44]	Canada	Cohort	ADT Orchiechtomy	Diabetes Acute myocardial infarction Sudden death	19,079 on ADT /Orchiechtomy 19,079 men with PCa no ADT	Increased risk of diabetes HR 1.16 (95%CI: 1.11–1.21). No increased risk of AMI HR 0.91 (95%CI: 0.84–1.00) or of sudden death HR 0.96 (95%CI: 0.83–1.10)

^1^ MetS Definition of the National Cholesterol Education Programme—Adult treatment panel III

^2^ Mets Definition of the International Diabetes Federation.

**Fig 2:**
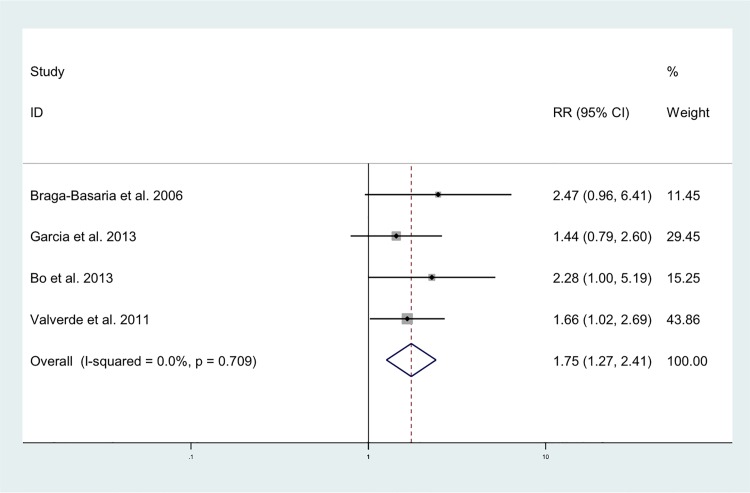
Forest plot for association between ADT and MetS.

**Fig 3:**
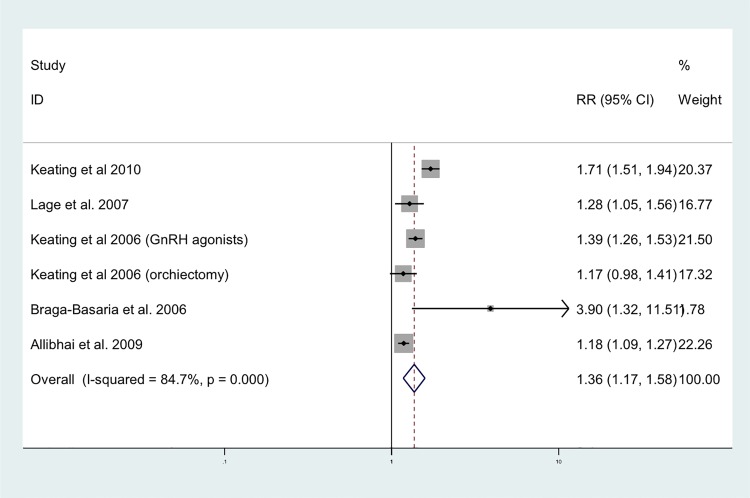
Forest plot for association between ADT and diabetes.

**Fig 4:**
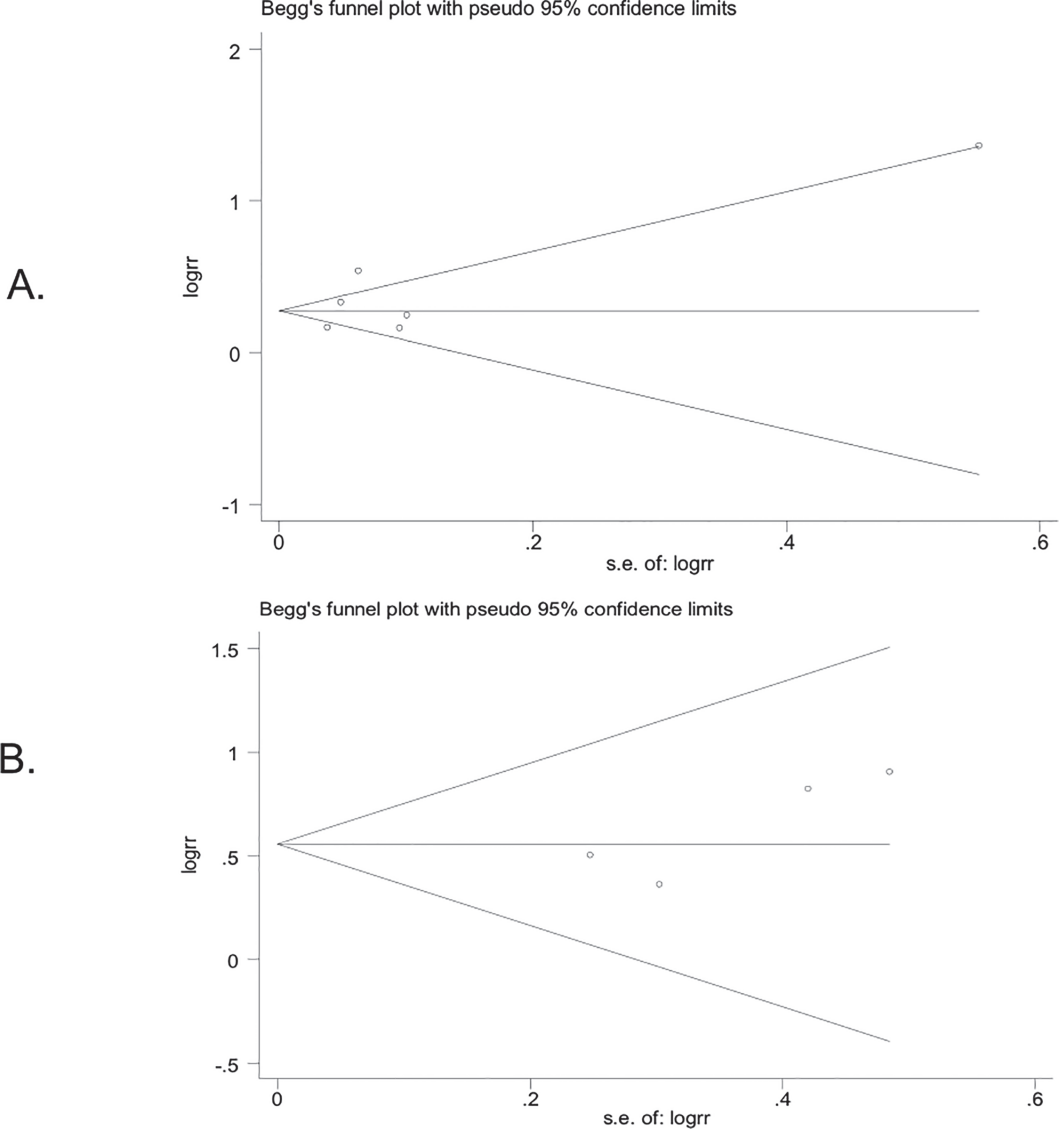
Begg’s funnel plots to test for publication bias for the associations between ADT and diabetes (a) and ADT and diabetes (b).

The random effects analysis, comparing ADT and risk of MetS, indicated a pooled effects relative risk of 1.75 (95%CI: 1.27–2.41). This pooled analysis included four studies of which three used the National Education Cholesterol Programme definition for MetS and one used the definition from the International Diabetes Federation [22,23]. The I^2^ statistic suggested no heterogeneity (I^2^ = 0.00%), which can also be observed in the corresponding Forest plot (Fig. 2). The only MetS component with more than 3 studies was diabetes. The random effects analysis showed a pooled relative risk of 1.36 (95%CI: 1.17–1.58) for the association between ADT and risk of diabetes. The I^2^ statistic suggested heterogeneity (I^2^ = 84.7%), but this was rather limited as can be seen in the corresponding Forest plot (Fig. 3). For both analyses, Begg’s test did not indicate publication bias (P: 0.34 and 0.23, respectively), which is also evident from the funnel plots as there is a symmetric distribution observed among studies (Fig. 4).

## Discussion

Despite the plethora of reviews on risk of MetS or its components following ADT for PCa [11–13,24], this is the first meta-analysis of evidence published to date. The results suggest a 75% increased risk of MetS and a 36% increased risk of diabetes following ADT for men with PCa.

In 2010, the Food and Drug Administration required labeling on gonadotropin-releasing hormone (GnRH) agonists, warning men about an increased risk of diabetes when receiving these medications for PCa treatment [25]. Supporting evidence came from large North- American cohorts [6,26,27]. To our knowledge no large European studies have yet quantified the risk of diabetes following ADT, nor have most cohorts made a distinction between different types and duration of ADT while comparing with PCa-free men. This information would be of interest to patients and clinicians as the association between ADT as PCa may be affected by differences in lifestyle and treatment practices, but most importantly by type and duration of ADT.

The current meta-analysis thus aimed to quantitatively summarize how ADT increases risk of metabolic abnormalities. The findings corroborated the above-mentioned FDA statement as we found a positive association for ADT with both MetS and diabetes. A recent systematic review, not including any summary statistics, also concluded that most studies indicate that ADT is positively associated with risk of insulin resistance and MetS [28]. However, based on the limitations of some studies, such as lack of sample size calculations or control groups, this review concluded that more studies are needed to further disentangle the association between ADT and metabolic abnormalities. The latter would require studies focused on specific subtypes of ADT, but most studies to date did not show results for different subtypes of ADT. The study by Keating et al. [6] was the only making a distinction between orchiectomy and GnRH agonists and found a slightly higher risk for those on GnRH agonists (HR: 1.44) than those who underwent orchiectomy (HR: 1.34). In a more recent study, the same authors showed that treatment with GnRH agonists was associated with increased risk of diabetes (HR: 1.28), but no statistically significant association was found for anti-androgens, combined androgen blockage, or orchiectomy [26]. Thus, even though most studies found a positive association between ADT and metabolic abnormalities, more experimental and epidemiological studies are needed to differentiate the metabolic effects of different types of ADT.

In addition to an increase in epidemiological evidence, also experimental biological findings are increasingly supporting an association between ADT and MetS or its components. Given that ADT lowers testosterone levels, evidence suggests that pathogenic mechanisms linking these low testosterone levels with MetS or its components are complex and bi-directional. Visceral obesity has been shown to be a cause of hypogonadism given that adipose tissue is a key source of estrogens due to the presence of an enzyme that converts testosterone into estrogens [29–32]. On the other hand, several studies have shown that testosterone increases lean body mass and that low levels of this hormone promote fat deposition [12,18,33]. Obesity has also been shown to be a major risk determinant for insulin resistance and type 2 diabetes [34]. Furthermore, low levels of testosterone have been linked to alterations in lipoprotein lipase enzyme activity and increases in triglyceride turnover, leading to abnormal levels of LDL and triglycerides, both components the MetS [35]. Additionally, testosterone has been suggested to regulate cell lineage determination by promoting the myogenic and inhibiting the adipogenic lineage [36].

Nonetheless, a limitation of observational studies is the possibility of bias introduced by selection of men to receive ADT. Men who are treated with ADT may differ from men who are not in ways that are also associated with risk for metabolic abnormalities. Although most of the studies included in this meta-analysis conducted analyses adjusting for observed confounders, we could only rely on crude event rates, as most studies did not provide sufficient data to allow us to account for potential confounders in our analyses. In future studies it would be of interest to add sensitivity analyses focused on specific subgroups of patients such as, for instance, those with or without a history of cardiovascular disease.

Our literature search methods were performed to include all relevant publications available to date through various sources, including grey literature, and two main online databases (Pubmed and Embase). Furthermore, objective inclusion and exclusion criteria were defined *a priori*. All included studies fulfilled these criteria and had a clearly defined study design and statistical analysis plan. There were however not enough studies available to examine the association between ADT and each component of MetS individually. Combining different definitions of MetS was needed given the small number of studies published to date and the recently published joint statement of major international associations defining everybody with three of the above listed metabolic risks as having MetS. However, all definitions were clearly stated and complied with this joint statement [14]. Furthermore, most studies investigated GnRH agonists as the main type of ADT, so that it was not possible to make a distinction between different types of ADT. Another limitation is that we could not make a distinction between patients with and without a history of cardiovascular disease. This difference could have shown whether ADT increases even more the risk of metabolic abnormalities in a subgroup of patients with cardiovascular history.

## Conclusion

This meta-analysis quantified the positive association between ADT for PCa and risk of developing MetS and diabetes. It also highlights the need to further disentangle this association by investigating different types and duration of ADT in relation to risk of these metabolic disturbances. The latter may provide insight in potential underlying mechanisms and may advocate the potential value of applying appropriate lifestyle changes.

## Supporting Information

S1 Checklist PRISMA Preferred Reporting Items for Systematic Reviews and Meta-Analyses checklist. (PDF)

S2 Checklist STROBE STrengthening the Reporting of OBservational studies in Epidemiology checklist. (PDF)
